# Structure and practices in hospitals of the Apice ON Project: a baseline study

**DOI:** 10.11606/s1518-8787.2020054001497

**Published:** 2020-02-05

**Authors:** Yluska Myrna Meneses Brandão e Mendes, Daphne Rattner

**Affiliations:** I Universidade de BrasíliaFaculdade de Ciências da SaúdeDepartamento de Saúde ColetivaBrasíliaDFBrasilUniversidade de Brasília. Faculdade de Ciências da Saúde. Departamento de Saúde Coletiva. Brasília/DF, Brasil; II Ministério da SaúdeSecretaria de VigilânciaBrasíliaDFBrasilMinistério da Saúde. Secretaria de Vigilância. Brasília/DF, Brasil

**Keywords:** Midwifery, Hospitals, Maternity, Schools, Health Occupations, Teaching Hospitals, Quality of Health Care, Maternal-Child Health Services

## Abstract

**OBJECTIVE:**

To describe some characteristics of the 97 teaching hospitals participating in the *Projeto de Aprimoramento e Inovação no Cuidado e Ensino em Obstetrícia e Neonatologia* (Apice ON—Project for Improvement and Innovation in Care and Teaching in Obstetrics and Neonatology).

**METHODS:**

The semester prior to the beginning of the program was adopted as the baseline to evaluate the subsequent structural and processes changes of this project. Secondary data from the first half of 2017 were extracted from the National Registry of Health Establishments (NRHE), the Hospital Information System and the *Sistema de Informações sobre Nascidos Vivos* (SINASC—Live Birth Information System).

**RESULTS:**

Before the implementation of the project, only 66% of the hospitals had a Baby-friendly Hospital Initiative, only 3% offered special accommodations for high-risk pregnant women, mothers and their newborns, and 45.4% hospitals adopted the skin-to-skin contact; 97% hospitals had separate rooms for pre-labor and vaginal delivery (93%), not following the recommendations of the Ministry of Health; nine hospitals (9%) had no rooming-in; there were few obstetrics nurses (less than 1% of professionals enrolled in the NRHE), and in only six hospitals the proportion of births assisted by this professional was above 50% of vaginal deliveries, while in eight this percentage ranged between 15% and 50%; the average cesarean section rate was 42%, ranging between 37.6% (Southeast) and 49.1% (Northeast); ten hospitals did not charge for companions according to inpatient hospital authorization.

**CONCLUSION:**

The study strengthens the relevance of the Apice ON project as an inducer of change of the care model in teaching hospitals and, therefore, as a strategy for the implementation of the national public policy represented by the Stork Network.

## INTRODUCTION

In August 2017, the Brazilian Ministry of Health (MH) implemented the *Projeto de Aprimoramento e Inovação no Cuidado e Ensino em Obstetrícia e Neonatologia *(Apice ON—Project for Improvement and Innovation in Care and Teaching in Obstetrics and Neonatology), aiming to promote changes in the model of care training of these specialties, as well as in the management of care processes in teaching hospitals (TH). The purpose of this project is to integrate the affiliate institutions – which define de learning of future professionals – to the Stork Network to incorporate attitudes and practices in harmony with care models. The proposed change encompasses practices based on scientific evidence and humanization of care processes in perinatal care^1^. Thus, it supports changes in the behavior and understanding of professionals who attend deliveries and births, in order to improve future professional practice.

### Brief background

Advances in the field of maternal health occurred in the 20th century. Firstly, perinatal care occurred at home, performed by midwives. With the institutionalization of childbirth in hospitals, more interventions were adopted to provide care, forming thus a progressively depersonalized care model, which is characterized by excessive interventions and disregard for the physiological, emotional and cultural aspects of birth. In Brazil, in the late 20th century, more than 90% of the deliveries were performed in hospitals^2^. Annually, three million births occur^3^, being 98.5% of these in hospitals^4^. In the early 1990s, advocates of humanized birth and birth care discussed the inadequacy of the existing care model, system characterized by excessive interventions, most of them without any scientific evidence and disrespectful of women’s sexual and reproductive rights^5^.

Davis-Floyd^6^ identified three care birth models, which differ regarding body definition and its relationship with the mind. The technocratic model—prevalent in the Western world—emphasizes the institutionalization of births, uncritical adoption of new technologies and interventionist attitude, with a mechanistic view of the birth process, represented by the metaphor “engine-object-path” (the engine being the uterus, the object, the fetus, and the vagina, the path)^2^. In this model, woman and baby are objects of care. The humanistic model—adopted in several European and Eastern countries—respects the physiology of the process, being concerned with the well-being of the newborn and woman all along the childbirth period and recognizing the body as an organism and women as a subject. The holistic model integrates the spiritual issue^6^, generally adopted by individual assistance providers. In Brazil, the technocratic model has been predominant, it is characterized by the use of a set of medical techniques, subjecting women to norms and routines that superimpose their physiological rhythm. One of the effects of this model is the high rate of births by surgical procedures^7^.

The current obstetrics and neonatal care policy represents a convergence of previous initiatives in the search for the care model based on appropriate technologies for humanized practices and attitudes. Initiatives aimed at changes to qualify the care provided to women in the pregnancy-puerperal cycle stand out, such as: the *Programa de Humanização do Pré-natal e Nascimento* (Prenatal and Birth Humanization Program—2000), which aimed to increase access to prenatal care, to guarantee beds for delivery and to improve the general structure of services^8^; the *Pacto Nacional pela Redução da Taxa de Cesárea* (National Pact for the Reduction of the Cesarean Section Rate—2000), delegating to states the control and monitor this rate^9^; the *Política Nacional de Humanização* (National Humanization Policy—2003), aiming at changes in health care and management models^10^; the *Pacto Nacional pela Redução da Mortalidade Materna e Neonatal* (National Pact for the Reduction of Maternal and Neonatal Mortality—2004); and SN (2011), proposing changes in the obstetrics care model, based on the best scientific evidence, with the aim to rescue the natural birth process^11^.

### About the Stork Network and the Apice ON Project

SN was implemented to modify the assistance in services and to promote the adequacy of birth care, gradually migrating to the humanistic model; however, the technocratic model continued to be adopted in teaching hospitals; as a result, the professionals educated at these hospitals reproduce the experiences and knowledge acquired, re-editing an inadequate model. This circumstance led the Ministries of Health and Education to develop a proposal for a change in the model of care in force in TH: the Apice ON project, established in a network of hospitals with teaching activities in order to implement and disseminate good practices, promoting changes in traditional models of training, care, and management.

Structure, combined with the process and the outcome, measures the health care quality^12^. The structure encompasses material, financial, and human resources, as well as equipment, available inputs, and organizational structure.

Therefore, this article aims to contribute to the Apice ON Project by describing structural and some process characteristics of the participating TH, adopting as the baseline the semester prior to the beginning of the program, so that the structural changes, practices and impacts arising from this project can be evaluated in the future.

## METHODS

This is a descriptive study on the characteristics of hospitals that are members of Apice ON, based on secondary data available from the National Register of Health Establishment(NRHE), the Hospital Information System (HIS/SUS) and the *Sistema de Informações sobre Nascidos Vivos* (SINASC—Brazilian Live Birth Information System), referring to the first half of 2017, before the beginning of the project.

A total of 97 certified teaching hospitals participating in the SN, from all regions of Brazil, joined Apice ON. In order to be included in the project, the hospitals, in addition to being TH, needed to have assisted more than 1,000 deliveries in 2015 according to HIS/SUS^1^.

Data on hospital production, per month and date of hospitalization (between January and June 2017), were extracted from the HIS/SUS database, whereas data on the structure of hospitals were obtained from NRHE. Data on births were extracted, per month of birth, from SINASC.

Microsoft Excel 2016 was used for analysis, with results presented in absolute numbers, means, proportions and Pearson’s chi-square test (χ^2^) with significance level of p < 0.05. Hospitals were stratified by their insertion in the regions of the country.

According to each information system, the variables of interest were:

**NRHE:** adult and/or neonatal beds of intensive care unit (ICU); teaching activity; reference hospital; legal nature; minimum team (obstetrician-gynecologist, nurse, obstetrics nurse, pediatrician, anesthesiologist, nursing technician, and nursing assistant); physical structure; equipment for mother and newborn care; services; qualifications; beds in obstetrics; physical installations.

**HIS:** vaginal birth (VB); VB in high-risk pregnancy; VB in VB Birth center; cesarean section (CS) in high-risk pregnancy; CS; CS with tubal ligation; post-abortion/puerperal curettage; overnight stay payment for pregnant women’s companion; treatment for postpartum complications; treatment for eclampsia, edema, proteinuria and hypertensive disorder in pregnancy, birth and postpartum period; treatment for clinical complications in pregnancy; surgical treatment for ectopic pregnancy; molar pregnancy; placental abruption; manual postpartum uterine inversion; treatment of other maternal pregnancy-related disorders.

**SINASC:** number of live births; type of birth; professional who assisted in the birth; birth weight.

The categorization of variables was: “quantity of deliveries in the semester”: up to 499 deliveries, between 500 and 999 and 1,000 or more deliveries (adapted from a similar publication^13^); “proportion of vaginal deliveries assisted by nurses or obstetrician”: none (0), between 0.01% and 5%, between 5% and 15%, between 15% and 50%, and above 50%; “cesarean section rate”: up to 30%, between 30% and 40%, between 40% and 50%, between 50% and 60%, and above 60%; “low birth weight ratio”: < 10%, between 10% and 15%, between 15% and 20%, and > 20%.

Considering this study used secondary databases of public domain, there was no need for appreciation by the Research Ethics Committee according to Resolution No. 510/2016 of the National Health Council.

## RESULTS

Table 1 describes the distribution of total hospitals and the project per regions of the country. Out of the 5,012 hospitals in Brazil, 97 (1.9%) are part of the project. We noted that 32.3% of hospitals in Brazil are located in the Northeast; the percentage is 17.5% among those in the project. The Southeast presents 38.1% of the total number of hospitals participating in the project, whereas the overall percentage in Brazil is 28.7%. The South concentrates 15.7% of the total number of the country’s hospitals and 18.6% of the project. The Midwest and the North had the lowest percentage of hospitals in Brazil, with 11.5% and 11.8%, respectively, and a distribution of 12.4% and 13.4% of the project.

**Table 1:** Distribution of hospitals participating in the Apice ON project and of hospitals in Brazil by region and Federal unit in 2017.

	Apice ON		Brazil	
	
	N	%	N	%
Midwest	12	12.4	574	11.5
Distrito Federal	4	4.1	25	0.5
Goiás	3	3.1	291	5.8
Mato Grosso do Sul	3	3.1	108	2.2
Mato Grosso	2	2.1	150	3.0
Northeast	17	17.5	1.619	32.3
Alagoas	1	1.0	68	1.4
Bahia	2	2.1	487	9.7
Ceará	3	3.1	220	4.4
Maranhão	1	1.0	248	4.9
Paraíba	2	2.1	88	1.8
Pernambuco	4	4.1	249	5.0
Piauí	1	1.0	114	2.3
Rio Grande do Norte	2	2.1	96	1.9
Sergipe	1	1.0	49	1.0
North	13	13.4	592	11.8
Acre	2	2.1	23	0.5
Amazonas	4	4.1	101	2.0
Amapá	1	1.0	24	0.5
Pará	2	2.1	278	5.5
Rondonia	2	2.1	76	1.5
Roraima	1	1.0	22	0.4
Tocantins	1	1.0	68	1.4
Southeast	37	38.1	1.439	28.7
Espírito Santo	2	2.1	72	1.4
Minas Gerais	11	11.3	489	9.8
Rio de aneiro	3	3.1	195	3.9
São Paulo	21	21.6	683	13.6
South	18	18.6	788	15.7
Paraná	6	6.2	357	7.1
Rio Grande do Sul	8	8.2	259	5.2
Santa Catarina	4	4.1	172	3.4

Total	97	100.0	5.012	100.0

Source: MS/SVS/DANTPS/CGIAE/SINASC.

Note: According to Pearson’s chi-square test (χ^2^), the difference in proportions between regions and establishments (Apice ON and not Apice ON) was statistically significant (p = 0.032).

Table 2 shows some structural characteristics of the hospitals participating in the project. It should be noted that features such as qualifications, physical installation and services can be cumulative. We observed that 18.6% of the hospitals were not classified as TH and 66% of these present the Baby-friendly Hospital Initiative qualification, with the highest proportions in the Northeast, North and South (88.2%, 84.6% and 77.8%, respectively). In relation to the *Casa da Gestante, Bebê e Puérpera* (a special facility for high-risk pregnant women, mothers and their newborns), only three hospitals are qualified, one in the North and two in the Southeast. Only two hospitals have qualification for in-hospital Birth Center, both in the Northeast. There are 44 (45.4%) qualified hospitals for neonatal care by the kangaroo care, 8 in the North, 8 in the Northeast, 15 in the Southeast, 9 in the South and 4 in the Midwest. A total of 87 (89.7%) hospitals present surgical obstetrics beds and neonatal ICU, 17 (100%) in the Northeast, 17 (94.4%) in the South, 12 (92.3%) in the North, 32 (86.5%) in the Southeast, and 9 (75.0%) in the Midwest, evidencing their characteristic of reference for high-risk situations. Regarding physical installation, 94 (96.9%) hospitals have labor rooms and 90 (92.8%) vaginal birthrooms. A total of 81 (83.5%) hospitals have human breast milk bank service, with the lowest percentage found in the North (76.9%). Among human resources, only 0.9% of the staff is composed of obstetrics nurses, whereas 5.9% is composed of obstetrician-gynecologists.

**Table 2:** Distribution and structural characteristics of hospitals participating in the Apice ON project by region and in Brazil between January and June 2017.

Structural characteristics	Midwest		Northeast		North		Southeast		South		Brazil	
					
	N	%	N	%	N	%	N	%	N	%	N	%
Teaching hospital												
Teaching hospital with teaching activity	7	58.3	8	47.1	2	15.4	29	78.4	15	83.3	61	62.9
Teaching hospital	2	16.7	7	41.2	0	0.0	7	18.9	2	11.1	18	18.6
Not classified	3	25.0	2	11.8	11	84.6	1	2.7	1	5.6	18	18.6
Clientele												
Spontaneous demand	0	0.0	1	5.9	1	7.7	2	5.4	0	0.0	4	4.1
Spontaneous and referenced demand	11	91.7	16	94.1	10	76.9	33	89.2	18	100.0	88	90.7
Referenced demand	1	8.3	0	0.0	2	15.4	2	5.4	0	0.0	5	5.2
Legal Nature:												
Federal	4	33.3	8	47.1	1	7.7	6	16.2	5	27.8	24	24.7
State	5	41.7	6	35.3	9	69.2	13	35.1	4	22.2	37	38.1
Municipal	1	8.3	1	5.9	2	15.4	5	13.5	0	0.0	9	9.3
Private	2	16.7	2	11.8	1	7.7	13	35.1	9	50.0	27	27.8
Qualifications												
Baby-friendly hospital incentive	5	41.7	15	88.2	11	84.6	19	51.4	14	77.8	64	66.0
Reference for secondary care to high-risk pregnancy	2	16.7	3	17.6	3	23.1	7	18.9	2	11.1	17	17.5
Reference for tertiary care to high-risk pregnancy	5	41.7	12	70.6	2	15.4	19	51.4	4	22.2	42	43.3
Reference for high-risk pregnancy type I	2	16.7	6	35.3	6	46.2	11	29.7	7	38.9	32	
* Casa da Gestante, Bebê e Puérpera*	0	0.0	0	0.0	1	7.7	2	5.4	0	0.0	3	3.1
Vaginal intra-hospital birth center type II LDR	0	0.0	2	11.8	0	0.0	0	0.0	0	0.0	2	2.1
Ligature	12	100.0	14	82.4	6	46.2	29	78.4	14	77.8	75	77.3
Conventional intermediate care unit (CICU)	6	50.0	15	88.2	11	84.6	22	59.5	12	66.7	66	68.0
Kangaroo intermediate care unit (KICU)	4	33.3	8	47.1	8	61.5	15	40.5	9	50.0	44	45.4
Neonatal ICU II	2	16.7	1	5.9	0	0.0	2	5.4	3	16.7	8	8.2
Adult ICU I	0	0.0	2	11.8	0	0.0	1	2.7	0	0.0	3	3.1
Beds												
Surgical obstetrics and neonatal ICU	9	75.0	17	100.0	12	92.3	32	86.5	17	94.4	87	89.7
Surgical obstetrics	1	8.3	0	0.0	1	7.7	0	0.0	1	5.6	3	3.1
Neonatal ICU	1	8.3	0	0.0	0	0.0	4	10.8	0	0.0	5	5.2
Not classified	1	8.3	0	0.0	0	0.0	1	2.7	0	0.0	2	2.1
Physical installation												
Pediatric service room	8	66.7	4	23.5	2	15.4	20	54.1	10	55.6	44	45.4
Women's rest/observation room	5	41.7	9	52.9	5	38.5	21	56.8	10	55.6	50	51.5
Consultation rooms	10	83.3	12	70.6	9	69.2	31	83.8	14	77.8	76	78.4
Labor room	12	100.0	17	100.0	12	92.3	35	94.6	18	100.0	94	96.9
Vaginal delivery room	10	83.3	17	100.0	13	100.0	32	86.5	18	100.0	90	92.8
Operating room	12	100.0	12	70.6	8	61.5	29	78.4	13	72.2	74	76.3
Joint accommodation beds	10	83.3	15	88.2	12	92.3	35	94.6	16	88.9	88	90.7
Equipment												
Doppler Ultrasound	10	83.3	17	100.0	12	92.3	36	97.3	16	88.9	91	93.8
Ultrasound	10	83.3	15	88.2	6	46.2	29	78.4	15	83.3	75	77.3
Conventional ultrasound	4	33.3	9	52.9	9	69.2	15	40.5	5	27.8	42	43.3
Services												
Reproductive health care service	10	83.3	12	70.6	9	69.2	29	78.4	15	83.3	75	77.3
Prenatal, birth and birth care service	11	91.7	17	100.0	12	92.3	34	91.9	18	100.0	92	94.8
Imaging diagnostic service	12	100.0	17	100.0	13	100.0	36	97.3	18	100.0	96	99.0
Physiotherapy service	12	100.0	16	94.1	12	92.3	30	81.1	17	94.4	87	89.7
Human milk bank	11	91.7	14	82.4	10	76.9	31	83.8	15	83.3	81	83.5

Total number of hospitals participating in Apice ON	12	100.0	17	100.0	13	100.0	37	100.0	18	100.0	97	100.0

Human resources												
Nurse	1.647	19.6	3.285	20.6	1.016	15.3	7.329	18.0	3.903	20.2	17.180	18.8
Obstetrics nurse	81	1.0	172	1.1	210	3.2	291	0.7	69	0.4	823	0.9
Pediatrician	700	8.3	1.385	8.7	446	6.7	3.054	7.5	971	5.0	6.555	7.2
Anesthesiologist	390	4.6	545	3.4	144	2.2	1.781	4.4	977	5.0	3.836	4.2
Pediatric surgeon	63	0.7	98	0.6	20	0.3	264	0.6	112	0.6	556	0.6
Obstetrician-gynecologist	510	6.1	1.309	8.2	609	9.2	2.104	5.2	848	4.4	5.379	5.9
Nurse technician	3.233	38.4	5.881	36.9	3.369	50.7	12.778	31.3	7.932	41.0	33.193	36.4
Nursing assistant	1.798	21.4	3.269	20.5	836	12.6	13.207	32.4	4.538	23.5	23.648	25.9

Total average of professionals per region	8.422	100.0	15.943	100.0	6.649	100.0	40.808	100.0	19.349	100.0	91.170	100.0

Source: CNES/DATASUS/CGSI.

LDR: labor, delivery and recovery room

Note: For human resources, the mean of the semester was calculated.

Table 3 shows the obstetrics procedures performed in these hospitals, which presented 46.0% of normal births, 33.2% cesarean section and 6.9% of post-abortion/puerperal curettage, being the highest proportion of this last variable (10.0%) found in the North and the lowest (4.7%) in the Midwest. Ten of these hospitals (10.3%) did not register the payment for companions in the inpatient hospital authorization (IHA), 5 of them in the Southeast.

**Table 3:** Distribution of obstetrics procedures performed in hospitals participating in the Apice ON project in Brazil and regions between January and June 2017.

Procedures	Midwest		Northeast		North		Southeast		South		Brazil	

	N	%	N	%	N	%	N	%	N	%	N	%
Treat. of complications of the postpartum period	793	4.2	702	1.6	931	2.0	538	0.8	261	0.8	3.225	1.5
Treatment for eclampsia	14	0.1	19	0.0	2	0.0	28	0.0	17	0.1	80	0.0
Treat. of edema, proteinuria, HTN. dis. PG. deliveries postpartum.	72	0.4	595	1.3	496	1.1	624	0.9	143	0.4	1.930	0.9
Treat. of clinical complications in pregnancy	2.407	12.7	4.921	11.0	4.562	10.0	5.684	8.5	3.956	12.4	21.530	10.3
Vaginal birth	6.604	34.9	9.182	20.4	17.447	38.2	24.739	37.0	11.087	34.7	69.059	33.2
Vaginal birth in high-risk gestation	800	4.2	8.924	19.9	2.992	6.6	8.076	12.1	4.140	12.9	24.932	12.0
Vaginal birth in Birth center (NDC)	0	0.0	313	0.7	2	0.0	1.321	2.0	0	0.0	1.636	0.8
Cesarean section in high-risk gestation	1.853	9.8	12.105	26.9	5.493	12.0	10.385	15.5	5.298	16.6	35.134	16.9
Cesarean section	4.831	25.5	4.106	9.1	8.293	18.2	8.737	13.1	4.334	13.6	30.301	14.5
Cesarean section with tubal ligation	454	2.4	639	1.4	553	1.2	1.474	2.2	601	1.9	3.721	1.8
Post-abortion/puerperal curettage	891	4.7	2.578	5.7	4.584	10.0	4.478	6.7	1.896	5.9	14.427	6.9
Others	208	1.1	833	1.9	270	0.6	729	1.1	247	0.8	2.287	1.1

Total procedures	18.927	100.0	44.917	100.0	45.625	100.0	66.813	100.0	31.980	100.0	208.262	100.0

Overnight stays for companions												
Hospitals that charged SUS for overnight stays by companions	12	100.0	17	100.0	11	84.6	32	86.5	15	83.3	87	89.7
Overnight stays by companions	37.949	NA	112.744	NA	88.863	NA	90.146	NA	45.367	NA	375.069	NA

Total number of hospitals participating in Apice ON	12	100.0	17	100.0	13	100.0	37	100.0	18	100.0	97	100.0

Source: SIH/DATASUS/CGSI.

Treat.: treatment; HTN. dis. PG. deliveries. postpartum: hypertensive disorder in pregnancy, deliveries and postpartum period; NA: not applicable

Table 4 presents the characteristics of the deliveries assisted in these hospitals. A total of 75 (77.3%) hospitals have a large quantity of deliveries (above 1,000), especially in the North region, which has 12 of its 13 hospitals in this category. Regarding vaginal deliveries assisted by obstetric nurses, 85.6% of hospitals displayed 0% to 15% of their births, with 94.1% of hospitals in the Northeast in this interval. Only six hospitals (6.2%) showed percentage above 50% assisted by obstetric nurses, four in the North and two in the Southeast. For cesarean section rates, 32 hospitals (33.0%) are between 40% and 50%, 24 hospitals, 50% and 60%, and 15 are above 60%. Notably, 39 TH (40.2%) present percentages above 50%, with emphasis on 8 of the 12 hospitals (66.7%) in the Midwest region.

**Table 4:** Distribution of the characteristics of births assisted in hospitals participating in the Apice ON project in Brazil and regions between January and June 2017.

Characteristics	Midwest		Northeast		North		Southeast		South		Brazil	
					
	N	%	N	%	N	%	N	%	N	%	N	%
Number of deliveries												
Up to 499 deliveries	1	8.3	0	0.0	0	0.0	1	2.7	1	5.6	3	3.1
Between 500 and 999 deliveries	3	25.0	2	11.8	1	7.7	11	29.7	2	11.1	19	19.6
Over 1,000 deliveries	8	66.7	15	88.2	12	92.3	25	67.6	15	83.3	75	77.3
Vaginal deliveries assisted by obstetrics nurses												
Zero	2	16.7	0	0.0	1	7.7	15	40.5	8	44.4	26	26.8
Between 0.01% and 5%	5	41.7	13	76.5	5	38.5	16	43.2	8	44.4	47	48.5
Between 5% and 15%	3	25.0	3	17.6	2	15.4	0	0.0	2	11.1	10	10.3
Between 15 and 50%	2	16.7	1	5.9	1	7.7	4	10.8	0	0.0	8	8.2
More than 50%	0	0.0	0	0.0	4	30.8	2	5.4	0	0.0	6	6.2
Low birth weight												
Less than 10%	2	16.7	3	17.6	6	46.2	4	10.8	4	22.2	19	19.6
Between 10% and 15%	5	41.7	1	5.9	5	38.5	20	54.1	7	38.9	38	39.2
Between 15 and 20%	4	33.3	4	23.5	1	7.7	7	18.9	4	22.2	20	20.6
More than 20%	1	8.3	9	52.9	1	7.7	6	16.2	3	16.7	20	20.6
Rate of cesarean section												
Up to 30%	0	0.0	0	0.0	1	7.7	4	10.8	1	5.6	6	6.2
Between 30% and 40%	0	0.0	1	5.9	2	15.4	11	29.7	6	33.3	20	20.6
Between 40% and 50%	4	33.3	6	35.3	7	53.8	10	27.0	5	27.8	32	33.0
Between 50% and 60%	7	58.3	6	35.3	2	15.4	5	13.5	4	22.2	24	24.7
More than 60%	1	8.3	4	23.5	1	7.7	7	18.9	2	11.1	15	15.5
Total number of hospitals participating in Apice ON	12	100.0	17	100.0	13	100.0	37	100.0	18	100.0	97	100.0

Source: MS/SVS/DANTPS/CGIAE/SINASC.

Table 5 shows the rates of cesarean sections in the hospitals of the project compared with those of regular hospitals in Brazil in the first half of 2017. A total of 56.9% of all newborns in Brazil were delivered by cesarean section, with a higher proportion in the South and Midwest (64.0% and 63.9%, respectively) and the lowest in the North (47.3%). In the hospitals within the project, the rate of cesarean section was 42.0%, being higher in the Midwest (49.1%) and lower in the Southeast (37.6%), showing the great difference in surgical practices among the hospitals selected for the project compared with the regular hospitals in the country.

**Table 5:** Distribution of the cesarean section rate in hospitals participating in the Apice ON project in Brazil and regions between January and June 2017.

Procedure	Midwest		Northeast		North		Southeast		South		Brazil	

	N	%	N	%	N	%	N	%	N	%	N	%
Brazil												
Cesarean sections	70.017	63.9	183.645	49.3	54.967	47.3	326.066	60.5	116.077	64.0	750.772	56.9
Total number of deliveries	109.584	100.0	372.600	100.0	116.195	100.0	539.387	100.0	181.422	100.0	1.319.188	100.0
Apice ON												
Cesarean section	7.138	49.1	16.850	47.8	14.339	41.2	20.596	37.6	10.233	40.2	69.156	42.0
Total number of deliveries	14.542	100.0	35.269	100.0	34.780	100.0	54.732	100.0	25.460	100.0	164.783	100.0

Source: MS/SVS/DANTPS/CGIAE/SINASC.

Note: In the estimation of hospitals in Brazil, the hospitals that are part of the Apice ON Project were excluded. According to Pearson’s chi-square test (χ^2^), the difference in proportions between regions and type of birth was statistically significant for Brazil and Apice ON hospitals (p < 0.00001).

## DISCUSSION

This study aimed to describe the structure and some process indicators of the teaching hospitals participating in the Apice ON project, that play a fundamental role in changing the current model of obstetric and neonatal care to a model that has been built since the 2000s, which aims at a humanized care for women and newborns and is based on scientific evidence, and enables women to experience their delivery and the birth of their child as a natural process, as recommended by the World Health Organization (WHO)^14^.

TH are characterized as an extension of health facilities, performing teaching practices, being officially recognized as teaching facilities and providing medical care at tertiary level^15^. In Brazil, the Interministerial Ordinance No. 1006/MEC/MS redefines the role of TH to meet the needs of the population—including the humanization of health care, guided by the Brazilian National Humanization Policy—, implementing the comprehensive care^16^. A study conducted in 14 medical schools showed that 86% of the workload of practical internships occur in university hospitals and 14% in other establishments^17^. Therefore, these internships must be modeled, so that learning is in accordance with scientific evidence, as well as with national and international recommendations and regulations for good practice.

According to the SN, continuous assistance to parturient is expected in the humanistic model by using adequate technology, avoiding invasive interventions and techniques without indication; having as place of birth, in addition to hospitals, Birth centers; with the presence of obstetrics nurses responsible for monitoring labor without dystocia; and, in case of any complications, being given the possibility to refer the parturient to other professionals^11^.

Other central aspect of the proposal is the adoption of good care practices, among them, the guarantee of a companion chosen by the woman during labor, birth and recovery (LDR); democratic and participatory management practices in services; adequate ambience in the obstetrics and neonatology sectors, according to the Collegiate Board Resolution (CBR) No. 36/2008 of the Brazilian Health Regulatory Agency (ANVISA), with intra and peri-hospital Birth centers; ambience in obstetrics centers, with individualized environments for continued LDR care, without transferring the parturient to another environment; offer of general and specialized obstetrics and neonatal beds (ICU, kangaroo care and *Casa da Gestante, Bebê e Puérpera*); and Baby-friendly Hospital Initiative, strengthening the beginning of breastfeeding and offering rooming-in, among other practices^18,19^.

The results of this baseline study showed that most hospitals attend high-risk pregnancies, because almost 95% of them have neonatal ICU, and more than 80% have important qualifications. However, before the implementation of the project, the proportion of Baby-friendly Hospital Initiative among hospitals was relatively low (66%), although this program exists since 1994^20^; nine hospitals (9%) still did not offer rooming-in, which is recommended since the 1980s^21^; few hospitals offered kangaroo care (45.4%), whose first ordinance dates from 2000^22^; it was almost null the proportion of hospitals with *Casa da Gestante, Bebê e Puérpera* (3.1%); and most hospitals had separate rooms for labor (97%) and vaginal birth (93%) diverging from ANVISA’s RCB No. 36/2008^19^; there were few obstetrics nurses (1% of the total number of professionals registered in NRHE), and the proportion of deliveries assisted by them was higher than 50% of vaginal deliveries in only six hospitals, between 15% and 50% in eight hospitals; the mean rate of cesarean section was 42%, ranging between 37.6% (Southeast) and 49.1% (Northeast); moreover, although Law No. 11,108^23^ and Ordinance GM/BMS No. 2418—ordinance which regulates this Law—^24^ are both from 2005, ten hospitals did not charge for the companion’s overnight stay in IHA.

Therefore, although the SN was established in 2011, aiming to change the current model of care to the humanistic one, and although the Prenatal and Birth Humanization Program was established in 2000, we highlight that, in 2017, most TH still adopted and reproduced the ambience and practices of the technocratic model, based on the rates of cesarean section and on the low number of vaginal deliveries assisted by obstetrics nurses.

In 1996, WHO edited a publication^25^ that classified the care practices for vaginal birth into four categories, based on the scientific knowledge available at the time: A. useful practices that should be stimulated; B. clearly harmful or ineffective practices that should be eliminated; C. practices without sufficient evidence to support a clear recommendation that should be applied with caution until further research clarifies the issue; and D. practices often used inappropriately. These recommendations were updated in 2018, adopting a new format^14^.

In this study, we could not identify the adoption of practices related in the WHO publication of 1996, with information only about cesarean sections. In relation to the country, the Birth in Brazil survey indicated that, regarding good practices in women at regular risk, 44.3% of these women reported movement during labor (category A). In 74.9% of the deliveries, central venous catheter was used (category B), in 39.1% of deliveries, the amniotomy (category C) was performed, and in 36.4%, oxytocin (category C) was used. Regarding vaginal delivery, 91.7% births were performed in the lithotomy position (category B) and 53.5% underwent episiotomy (category D). As to the type of birth, 51.9% of deliveries were cesarean sections (category D) and only 5% had vaginal birth without any intervention during the entire labor^26^.

Regarding care practices for healthy newborns in Brazil, 71.0% had their upper airways aspirated, procedure not recommended in the 2018 update^14^. Only 28.2% of the newborns had skin-to-skin contact at birth (category A), 69% newborns were placed in rooming-in accommodation (category A), and only 44.5% newborns were breastfed in the first hour of life (category A)^27^.

We can assume that these national data are similar to those of TH. Thus, it is noteworthy the strategic role of teaching hospitals of changing the technocratic model of care to the humanistic one, because if teaching occurs in places that adopt the humanistic model, as well as scientifically based practices, professionals will be more likely to reproduce them in their future practices. Rattner^5^ (2009) considered paradoxical that, in Brazil, perinatal care practices based on scientific evidence, recommended by WHO and BMS, were not adopted in educational institutions, in contrast, they were one of the focuses of resistance to their adoption, since the academy is expected to be the site privileged for the advancement of knowledge and use of best practices. Hotimsky^28^—in a study on obstetrics training in two renowned medical schools—,identified how technical-scientific competence and care for women in theoretical-practical teaching of birth care are united. Among her various findings, the following are highlighted: doctors do not share some decisions with women; presence of informal agreements between teams for the shifts; predominance of oral transmission of technical-scientific knowledge, devaluing the transmission of humanistic values and disregarding recent scientific evidence to define practices.

Changes in the national curriculum guidelines of health disciplines have been proposed since 2001. Among the proposals for the biomedical model, the teaching of humanization for comprehensive care and improvement in care are included. However, only the insertion of contents and disciplines involving the theme do not guarantee significant changes in training, since these contents are dispersed in disciplines that communicate little with each other. Humanization is more than a content, because it encompasses philosophical aspects of training, teaching practices and professional attitudes in the health and education scenarios, which are configured as a model for future practice, since professors are models of social and professional role during graduation^29^.

The main limitations of this study derive from the use of secondary data, with the possibility of incompleteness, under-registration and inadequate recording of information. Possibly, some indicators do not reflect the reality of ambience, since not all institutions update their data periodically in NRHE. The same occurs to IHA and the live birth certificates, although their bias is probably lower, since HIS/SUS is used for billing and the SINASC variables used here present good completeness. Another limitation derives from the fact that Apice ON Project is new and unprecedent, thus, there is a lack of publications on similar projects to enhance this discussion. On the other hand, studies such as this one are essential because, when performing baseline diagnoses, they produce the necessary support to further evaluate the impact of Apice ON actions on TH, supporting future decisions. Moreover, secondary databases enable us to monitor these same indicators without larger investments.

Finally, the scenario of change is promising, since there is a great distance between what should be adopted and taught in these institutions and what this baseline portrait evidences. Davis-Floyd et al. (p. 452)^30^ claim:

Health care professionals tend to practice as they were taught, so much so that when new information is presented, many long-time practitioners refuse to integrate or implement it because they are so habituated to doing things the way their teachers did. A primary key to the creation and maintenance of birth models that work is the reform of professional education, so that instead of being educated in the technology- and pathology-oriented biomedical model approach to birth, student doctors, nurses, and midwives are educated in the humanistic and normality-oriented midwifery model of birth.

Hotimsky^28^ identified in 2007 that teaching in the institutions studied had not seized scientifically-based content and humanized practices of perinatal care yet. Based on these results, we can assume that the current scenario in TH in 2017 (i.e., more than 20 years after the WHO publication) remains unchanged, proving the relevance of Apice ON as a strategy to implement the public policy represented by SN, whose purpose is to induce change in the care model in TH. A similar future study is expected to identify major changes that might have occurred in these institutions, both in the ambience and other aspects of the structure, and in the care process, causing an impact on its results, but, with greater property, in the professional practice of health professionals.
