# Supplementary material for: Structure and practices in hospitals of the Apice ON Project: a baseline study
Source: Rev Saude Publica. 2020 Feb 5;54:23. doi: 10.11606/s1518-8787.2020054001497 (PMC7006912; doi:10.11606/s1518-8787.2020054001497)
Supplement: Translation

# Translation: Estrutura e práticas de hospitais integrantes do Projeto Apice ON: estudo de linha de base

**Authors:** Yluska Myrna Meneses Brandão e Mendes, Daphne Rattner

**Affiliations:** I BrasilUniversidade de Brasília. Faculdade de Ciências da Saúde. Departamento de Saúde Coletiva. Brasília/DF, Brasil; II BrasilMinistério da Saúde. Secretaria de Vigilância. Brasília/DF, Brasil

**Keywords:** Tocologia, Maternidades, Escolas para Profissionais de Saúde, Hospitais de Ensino, Qualidade da Assistência à Saúde, Serviços de Saúde Materno-Infantil

## Abstract

**OBJETIVO:**

Este artigo descreve algumas características dos 97 hospitais de ensino participantes do Projeto de Aprimoramento e Inovação no Cuidado e Ensino em Obstetrícia e Neonatologia (Apice ON).

**MÉTODOS:**

Foi adotado como linha de base o semestre anterior ao lançamento do programa, para permitir avaliar as mudanças estruturais e processuais decorrentes desse projeto. Utilizaram-se dados secundários referentes ao primeiro semestre de 2017 disponíveis no Cadastro Nacional de Estabelecimentos de Saúde (CNES), no Sistema de Informações Hospitalares e no Sistema de Informações sobre Nascidos Vivos.

**RESULTADOS:**

Antes da implantação do projeto, apenas 66% dos hospitais apresentaram habilitação de Hospital Amigo da Criança, somente 3% estavam habilitados com Casa da gestante, Bebê e Puérpera e 45,4% adotavam o método canguru; 97% dispunham de sala de pré-parto e 93% de sala de parto normal separadas, sem seguir o preconizado pelo Ministério da Saúde; nove hospitais (9%) não tinham alojamento conjunto; havia poucos enfermeiros obstetras (menos de 1% dos profissionais cadastrados no CNES), e em apenas seis hospitais a proporção de partos assistidos por esse profissional foi superior a 50% dos partos vaginais, enquanto em oito hospitais esta proporção ficou entre 15 e 50%; a taxa média de cesáreas foi de 42%, variando entre 37,6% (Sudeste) e 49,1% (Nordeste); em dez dos hospitais não constava cobrança de diária de acompanhante na autorização de internação hospitalar.

**CONCLUSÃO:**

O estudo fortalece a pertinência do projeto Apice ON como indutor de mudança do modelo nos hospitais de ensino e, portanto, como estratégico para a efetivação da política pública nacional representada pela Rede Cegonha.

## INTRODUÇÃO

Em agosto de 2017, o Ministério da Saúde (MS) instituiu o Projeto de Aprimoramento e Inovação no Cuidado e Ensino em Obstetrícia e Neonatologia (Apice ON), visando impulsionar movimentos de mudança no modelo de formação e atenção nessas especialidades, assim como na gestão dos processos de atenção em hospitais de ensino (HE). Esse projeto propõe integrar à Rede Cegonha (RC) as instituições aderidas, definidoras do aprendizado de futuros profissionais, para incorporação de atitudes e práticas em sintonia com modelos assistenciais. A mudança proposta engloba conceitos como práticas fundamentadas em evidências científicas e humanização dos processos de cuidado na atenção perinatal^1^. Assim, oportuniza mudanças no comportamento e compreensão dos profissionais que assistem partos e nascimentos, para impactar a formação com vistas à futura prática profissional.

### Breve histórico

Avanços no campo da saúde materna ocorreram no século XX. No início, a atenção perinatal ocorria no domicílio, por parteiras. Com a institucionalização do parto em hospitais, adotaram-se mais intervenções para prestação de cuidados, conformando um modelo de assistência progressivamente despersonalizado, que se caracteriza por excesso de intervenções e desconsideração dos aspectos fisiológicos, emocionais e culturais do ato de parir. No Brasil, final do século XX, mais de 90% dos partos eram realizados em hospitais^2^. Anualmente têm ocorrido cerca de três milhões de nascimentos^3^, sendo 98,5% em ambiente hospitalar^4^. No início dos anos 1990, militantes da humanização da atenção ao parto e nascimento debatiam a inadequação do modelo de assistência existente, caracterizado por excesso de intervenções, a maioria sem fundamentação em evidências científicas, e desrespeito aos direitos sexuais e reprodutivos das mulheres^5^.

Davis-Floyd^6^ identificou três modelos de cuidados de atenção ao parto, que se diferenciam em relação à definição do corpo e sua relação com a mente. O modelo tecnocrático, prevalente no mundo ocidental, ressalta a institucionalização do parto, adoção acrítica de novas tecnologias e atitude intervencionista, com visão mecanicista do processo de parir, representada pela metáfora “motor-objeto-trajeto” (o motor sendo o útero, o objeto o feto e o trajeto a vagina)^2^. Nesse modelo, mulher e bebê são objetos de cuidado. O modelo humanista, adotado em vários países europeus e orientais, respeita a fisiologia do processo, com preocupação pelo bem-estar da puérpera e do recém-nascido, e reconhece o corpo como um organismo e a mulher como sujeito. Já o modelo holístico integra a questão espiritual^6^, adotado, em geral, por prestadores de assistência individuais. No Brasil, o modelo predominante tem sido o tecnocrático, caracterizado pela aplicação de um conjunto de técnicas médicas, submetendo as mulheres a normas e rotinas que se sobrepõem ao seu ritmo fisiológico. Um de seus efeitos é a alta taxa de nascimentos por procedimentos cirúrgicos^7^.

A política atual de atenção obstétrica e neonatal representa uma convergência de iniciativas anteriores na busca do modelo de atenção baseado em tecnologias apropriadas para o parto e nascimento com práticas e atitudes humanizadas. Destacam-se iniciativas voltadas às mudanças para qualificar a atenção à mulher no ciclo gravídico-puerperal, tais como: o Programa de Humanização do Pré-natal e Nascimento (2000), que objetivou incrementar o acesso ao pré-natal, garantir leitos para o parto e melhorar a estrutura dos serviços^8^; o Pacto Nacional pela Redução da Taxa de Cesárea (2000), com o qual estados passaram a controlar e monitorar essa taxa^9^; a Política Nacional de Humanização (2003), objetivando mudanças nos modelos de atenção e gestão na saúde^10^; o Pacto Nacional pela Redução da Mortalidade Materna e Neonatal (2004); e a RC (2011), propondo mudanças no modelo de atenção obstétrica com práticas baseadas nas melhores evidências científicas, visando resgatar o processo natural do parto e nascimento^11^.

### Sobre a Rede Cegonha e o Projeto Apice ON

A RC foi instituída para mudar a assistência nos serviços e promover a adequação da atenção ao parto, gradualmente migrando para o modelo humanista; todavia, o modelo tecnocrático ainda persiste e continua sendo adotado nos HE; em decorrência, os profissionais ali formados reproduzem as vivências e os aprendizados adquiridos, reeditando um modelo inadequado. Tal circunstância levou os ministérios da saúde e da educação a elaborar uma proposta de mudança no modelo de atenção vigente em HE: o projeto Apice ON, instituído numa rede de hospitais com atividades de ensino para contribuir com a implementação e disseminação de boas práticas, promovendo mudanças nos modelos tradicionais de formação, atenção e gestão.

Um dos componentes da qualidade dos serviços é a estrutura que, aliada com o processo e o resultado, medem a qualidade da atenção em saúde^12^. Ela engloba os recursos materiais, financeiros e humanos, assim como equipamentos, insumos disponíveis e estrutura organizacional.

Portanto, este artigo objetiva contribuir para o projeto Apice ON descrevendo características estruturais e algumas processuais dos HE participantes, adotando como linha de base o semestre anterior ao lançamento do programa, para que seja possível posteriormente avaliar as mudanças estruturais, de práticas e os impactos decorrentes desse projeto.

## MÉTODOS

Trata-se de estudo descritivo sobre as características dos hospitais integrantes do Apice ON, com base em dados secundários disponíveis no Cadastro Nacional de Estabelecimentos de Saúde (CNES), no Sistema de Informações Hospitalares (SIH/SUS) e no Sistema de Informações sobre Nascidos Vivos (Sinasc), referentes ao primeiro semestre do ano de 2017, ou seja, anteriores ao lançamento do projeto.

Aderiram ao Apice ON 97 hospitais certificados como de ensino e aderidos à RC de todas as regiões do Brasil. Para inclusão no projeto, além de serem HE, deveriam ter assistido mais de 1.000 partos em 2015 de acordo com o SIH/SUS^1^.

Dados sobre a produção hospitalar, por mês e data de internação (de janeiro a junho de 2017), foram extraídos da base de dados do SIH/SUS, enquanto os dados da estrutura dos hospitais foram obtidos no CNES. Dados sobre nascimentos foram extraídos, por mês de nascimento, do Sinasc.

Para análise utilizou-se o Microsoft Excel versão 2016, com resultados apresentados em números absolutos, médias, proporções e teste do qui-quadrado de Pearson (χ^2^) com nível de significância de p < 0,05. Os hospitais foram estratificados por sua inserção nas regiões do país.

As variáveis de interesse, segundo cada sistema de informação, foram:

**CNES:** leitos de unidade de terapia intensiva (UTI) adulto e/ou neonatal; atividade de ensino; hospital de referência; natureza jurídica; equipe mínima (médico gineco-obstetra, enfermeiro, enfermeiro obstetra, pediatra, anestesista, auxiliar e técnico de enfermagem); estrutura física; equipamentos para o cuidado da mãe e neonatos; serviços realizados; habilitações; leitos em obstetrícia; instalações físicas.

**SIH:** parto normal (PN); PN em gestação de alto risco; PN em centro de PN; parto cesariano (PC) em gestação alto risco; PC; PC com laqueadura tubária; curetagem pós-abortamento/puerperal; diárias de acompanhantes de gestante com pernoite; tratamento para complicações do puerpério; tratamento para eclampsia, edema, proteinúria e transtorno hipertensivo na gravidez, parto e puerpério; tratamento para intercorrências clínicas na gravidez; tratamento cirúrgico para gravidez ectópica; mola hidatiforme; descolamento manual de placenta; inversão manual uterina pós-parto; tratamento de outros transtornos maternos relacionados à gravidez.

**Sinasc:** número de nascidos vivos; tipo de parto; profissional que assistiu ao parto; peso ao nascer.

A categorização das variáveis foi: “volume de partos no semestre”: até 499 partos, 500 a 999 e 1.000 ou mais partos (adaptada de publicação semelhante^13^); “proporção de partos vaginais assistidos por enfermeiro ou obstetriz”: nenhum (0), 0,01 a 5%, 5 a 15%, 15 a 50% e acima de 50%; “taxa de cesárea”: até 30%, 30 a 40%, 40 a 50%, 50 a 60% e acima de 60%; “proporção de baixo peso ao nascer”: < 10%, 10 a 15%, 15 a 20% e > 20%.

Considerando que o presente estudo utilizou bases de dados secundárias de domínio público, não houve necessidade de apreciação pelo comitê de ética em pesquisa, nos termos da Resolução nº 510/2016 do Conselho Nacional de Saúde.

## RESULTADOS

A Tabela 1 descreve a distribuição de hospitais total e do projeto por regiões do país. Dos 5.012 hospitais do Brasil, 97 (1,9%) fazem parte do projeto. Nota-se que 32,3% dos hospitais do Brasil estão no Nordeste; dentre os do projeto, a proporção é de 17,5%. Outra diferença é no Sudeste, onde há 38,1% dos hospitais do projeto, enquanto no Brasil a proporção é de 28,7%. O Sul concentra 15,7% dos hospitais do país e 18,6% do projeto. O Centro-Oeste e o Norte apresentaram o menor percentual de hospitais do Brasil, com 11,5% e 11,8% respectivamente, com distribuição de 12,4% e 13,4% do projeto.

**Tabela 1:** Distribuição dos hospitais integrantes do projeto Apice ON e dos hospitais do Brasil por região e unidade federativa em 2017.

	Apice ON		Brasil	
	
	N	%	N	%
Centro-Oeste	12	12,4	574	11,5
DF	4	4,1	25	0,5
GO	3	3,1	291	5,8
MS	3	3,1	108	2,2
MT	2	2,1	150	3,0
Nordeste	17	17,5	1.619	32,3
AL	1	1,0	68	1,4
BA	2	2,1	487	9,7
CE	3	3,1	220	4,4
MA	1	1,0	248	4,9
PB	2	2,1	88	1,8
PE	4	4,1	249	5,0
PI	1	1,0	114	2,3
RN	2	2,1	96	1,9
SE	1	1,0	49	1,0
Norte	13	13,4	592	11,8
AC	2	2,1	23	0,5
AM	4	4,1	101	2,0
AP	1	1,0	24	0,5
PA	2	2,1	278	5,5
RO	2	2,1	76	1,5
RR	1	1,0	22	0,4
TO	1	1,0	68	1,4
Sudeste	37	38,1	1.439	28,7
ES	2	2,1	72	1,4
MG	11	11,3	489	9,8
RJ	3	3,1	195	3,9
SP	21	21,6	683	13,6
Sul	18	18,6	788	15,7
PR	6	6,2	357	7,1
RS	8	8,2	259	5,2
SC	4	4,1	172	3,4

Total	97	100,0	5.012	100,0

Fonte: MS/SVS/DANTPS/CGIAE/Sinasc.

Nota: Pelo teste do qui-quadrado de Pearson (χ^2^), a diferença das proporções entre as regiões e estabelecimentos (Apice ON e não Apice ON) foi estatisticamente significante (p = 0,032).

Na Tabela 2 estão relacionadas algumas características estruturais dos hospitais do projeto. Saliente-se que características como habilitações, instalação física e serviços podem ser cumulativas. Observa-se que 18,6% dos hospitais não foram classificados como HE e que 66% possuem habilitação de Amigo da Criança, com as maiores proporções no Nordeste, Norte e Sul (88,2%, 84,6% e 77,8%, respectivamente). Em relação à Casa da Gestante, Bebê e Puérpera, apenas três são habilitados, um no Norte e dois no Sudeste. Apenas dois possuem habilitação para CPN intra-hospitalar, ambos no Nordeste. Há 44 (45,4%) habilitados para cuidados neonatais pelo método canguru, sendo 8 no Norte, 8 no Nordeste, 15 no Sudeste, 9 no Sul e 4 no Centro-Oeste. Quanto aos leitos de obstetrícia cirúrgica e UTI neonatal, 87 (89,7%) hospitais os possuem, sendo 17 (100%) no Nordeste, 17 (94,4%) no Sul, 12 (92,3%) no Norte, 32 (86,5%) no Sudeste e 9 (75,0%) no Centro-Oeste, evidenciando sua característica de referência para alto risco. Quanto à instalação física, 94 (96,9%) possuem salas de pré-parto e 90 (92,8%) salas de PN. Há 81 (83,5%) com serviço de banco de leite, sendo o menor percentual encontrado no Norte (76,9%). Dentre os recursos humanos, apenas 0,9% do quadro é composto por enfermeiros obstetras, enquanto 5,9% é formado por médicos gineco-obstetras.

**Tabela 2:** Distribuição e características estruturais dos hospitais integrantes do projeto Apice ON por região e no Brasil de janeiro a junho de 2017.

Características estruturais	Centro-Oeste		Nordeste		Norte		Sudeste		Sul		Brasil	
					
	N	%	N	%	N	%	N	%	N	%	N	%
Hospital de ensino												
Hospital de ensino com atividade de ensino	7	58,3	8	47,1	2	15,4	29	78,4	15	83,3	61	62,9
Hospital de ensino	2	16,7	7	41,2	0	0,0	7	18,9	2	11,1	18	18,6
Não classificado	3	25,0	2	11,8	11	84,6	1	2,7	1	5,6	18	18,6
Clientela												
Demanda espontânea	0	0,0	1	5,9	1	7,7	2	5,4	0	0,0	4	4,1
Demanda espontânea e referenciada	11	91,7	16	94,1	10	76,9	33	89,2	18	100,0	88	90,7
Demanda referenciada	1	8,3	0	0,0	2	15,4	2	5,4	0	0,0	5	5,2
Natureza jurídica												
Federal	4	33,3	8	47,1	1	7,7	6	16,2	5	27,8	24	24,7
Estadual	5	41,7	6	35,3	9	69,2	13	35,1	4	22,2	37	38,1
Municipal	1	8,3	1	5,9	2	15,4	5	13,5	0	0,0	9	9,3
Privada	2	16,7	2	11,8	1	7,7	13	35,1	9	50,0	27	27,8
Habilitações												
Amigo da Criança	5	41,7	15	88,2	11	84,6	19	51,4	14	77,8	64	66,0
Referência para atendimento secundário à gestação de alto risco	2	16,7	3	17,6	3	23,1	7	18,9	2	11,1	17	17,5
Referência para atendimento terciário à gestação de alto risco	5	41,7	12	70,6	2	15,4	19	51,4	4	22,2	42	43,3
Referência para gestação de alto risco tipo I	2	16,7	6	35,3	6	46,2	11	29,7	7	38,9	32	33,0
Casa da Gestante, Bebê e Puérpera	0	0,0	0	0,0	1	7,7	2	5,4	0	0,0	3	3,1
Centro de parto normal intra-hospitalar tipo II PPP	0	0,0	2	11,8	0	0,0	0	0,0	0	0,0	2	2,1
Laqueadura	12	100,0	14	82,4	6	46,2	29	78,4	14	77,8	75	77,3
Unidade de cuidados intermediários convencionais (Ucinco)	6	50,0	15	88,2	11	84,6	22	59,5	12	66,7	66	68,0
Unidade de cuidados intermediários canguru (Ucinca)	4	33,3	8	47,1	8	61,5	15	40,5	9	50,0	44	45,4
UTI II neonatal	2	16,7	1	5,9	0	0,0	2	5,4	3	16,7	8	8,2
UTI I adulto	0	0,0	2	11,8	0	0,0	1	2,7	0	0,0	3	3,1
Leitos												
Obstetrícia cirúrgica e UTI neonatal	9	75,0	17	100,0	12	92,3	32	86,5	17	94,4	87	89,7
Obstetrícia cirúrgica	1	8,3	0	0,0	1	7,7	0	0,0	1	5,6	3	3,1
UTI neonatal	1	8,3	0	0,0	0	0,0	4	10,8	0	0,0	5	5,2
Não classificado	1	8,3	0	0,0	0	0,0	1	2,7	0	0,0	2	2,1
Instalação física												
Sala de atendimento pediátrico	8	66,7	4	23,5	2	15,4	20	54,1	10	55,6	44	45,4
Sala de repouso/observação feminina	5	41,7	9	52,9	5	38,5	21	56,8	10	55,6	50	51,5
Consultórios médicos	10	83,3	12	70,6	9	69,2	31	83,8	14	77,8	76	78,4
Sala de pré-parto	12	100,0	17	100,0	12	92,3	35	94,6	18	100,0	94	96,9
Sala de parto normal	10	83,3	17	100,0	13	100,0	32	86,5	18	100,0	90	92,8
Sala de cirurgia	12	100,0	12	70,6	8	61,5	29	78,4	13	72,2	74	76,3
Leitos de alojamento conjunto	10	83,3	15	88,2	12	92,3	35	94,6	16	88,9	88	90,7
Equipamentos												
Ultrassom Doppler colorido	10	83,3	17	100,0	12	92,3	36	97,3	16	88,9	91	93,8
Ultrassom ecógrafo	10	83,3	15	88,2	6	46,2	29	78,4	15	83,3	75	77,3
Ultrassom convencional	4	33,3	9	52,9	9	69,2	15	40,5	5	27,8	42	43,3
Serviços												
Serviço de atenção à saúde reprodutiva	10	83,3	12	70,6	9	69,2	29	78,4	15	83,3	75	77,3
Serviço de atenção ao pré-natal, parto e nascimento	11	91,7	17	100,0	12	92,3	34	91,9	18	100,0	92	94,8
Serviço de diagnóstico por imagem	12	100,0	17	100,0	13	100,0	36	97,3	18	100,0	96	99,0
Serviço de fisioterapia	12	100,0	16	94,1	12	92,3	30	81,1	17	94,4	87	89,7
Banco de leite	11	91,7	14	82,4	10	76,9	31	83,8	15	83,3	81	83,5

Total de hospitais no Apice ON	12	100,0	17	100,0	13	100,0	37	100,0	18	100,0	97	100,0

Recursos humanos												
Enfermeiro	1.647	19,6	3.285	20,6	1.016	15,3	7.329	18,0	3.903	20,2	17.180	18,8
Enfermeiro obstetra	81	1,0	172	1,1	210	3,2	291	0,7	69	0,4	823	0,9
Médico pediatra	700	8,3	1.385	8,7	446	6,7	3.054	7,5	971	5,0	6.555	7,2
Médico anestesiologista	390	4,6	545	3,4	144	2,2	1.781	4,4	977	5,0	3.836	4,2
Médico cirurgião pediátrico	63	0,7	98	0,6	20	0,3	264	0,6	112	0,6	556	0,6
Médico ginecologista e obstetra	510	6,1	1.309	8,2	609	9,2	2.104	5,2	848	4,4	5.379	5,9
Técnico de enfermagem	3.233	38,4	5.881	36,9	3.369	50,7	12.778	31,3	7.932	41,0	33.193	36,4
Auxiliar de enfermagem	1.798	21,4	3.269	20,5	836	12,6	13.207	32,4	4.538	23,5	23.648	25,9

Média total de profissionais por região	8.422	100,0	15.943	100,0	6.649	100,0	40.808	100,0	19.349	100,0	91.170	100,0

Fonte: CNES/DATASUS/CGSI.

PPP: trabalho de parto, parto e pós-parto imediato

Nota: Para recursos humanos, foi calculada a média do semestre.

A Tabela 3 apresenta os procedimentos obstétricos realizados nesses hospitais, que apresentaram 46,0 % de PN, 33,2% de cesariana e 6,9% de curetagem pós-abortamento/puerperal, sendo a maior proporção desta (10,0%) encontrada no Norte e a menor (4,7%) no Centro-Oeste. Desses hospitais, 10 (10,3%) não registraram solicitação de pagamento de diárias para acompanhantes na autorização de internação hospitalar (AIH), 5 deles no Sudeste.

**Tabela 3:** Distribuição dos procedimentos obstétricos realizados nos hospitais integrantes do projeto Apice ON no Brasil e regiões de janeiro a junho de 2017.

Procedimentos	Centro-Oeste		Nordeste		Norte		Sudeste		Sul		Brasil	
	N	%	N	%	N	%	N	%	N	%	N	%
Trat. de complicações predominantes do puerpério	793	4,2	702	1,6	931	2,0	538	0,8	261	0,8	3.225	1,5
Trat. de eclampsia	14	0,1	19	0,0	2	0,0	28	0,0	17	0,1	80	0,0
Trat. de edema, proteinúria, trans. hip. grav. part. puerp.	72	0,4	595	1,3	496	1,1	624	0,9	143	0,4	1.930	0,9
Trat. de intercorrências clínicas na gravidez	2.407	12,7	4.921	11,0	4.562	10,0	5.684	8,5	3.956	12,4	21.530	10,3
Parto normal	6.604	34,9	9.182	20,4	17.447	38,2	24.739	37,0	11.087	34,7	69.059	33,2
Parto normal em gestação de alto risco	800	4,2	8.924	19,9	2.992	6,6	8.076	12,1	4.140	12,9	24.932	12,0
Parto normal em centro de parto normal (CPN)	0	0,0	313	0,7	2	0,0	1.321	2,0	0	0,0	1.636	0,8
Parto cesariano em gestação de alto risco	1.853	9,8	12.105	26,9	5.493	12,0	10.385	15,5	5.298	16,6	35.134	16,9
Parto cesariano	4.831	25,5	4.106	9,1	8.293	18,2	8.737	13,1	4.334	13,6	30.301	14,5
Parto cesariano com laqueadura tubária	454	2,4	639	1,4	553	1,2	1.474	2,2	601	1,9	3.721	1,8
Curetagem pós-abortamento / puerperal	891	4,7	2.578	5,7	4.584	10,0	4.478	6,7	1.896	5,9	14.427	6,9
Outros	208	1,1	833	1,9	270	0,6	729	1,1	247	0,8	2.287	1,1
Total de procedimentos	18.927	100,0	44.917	100,0	45.625	100,0	66.813	100,0	31.980	100,0	208.262	100,0
Diárias para acompanhantes												
Hospitais que pagam diárias para acompanhantes	12	100,0	17	100,0	11	84,6	32	86,5	15	83,3	87	89,7
Diárias pagas para acompanhantes	37.949	NSA	112.744	NSA	88.863	NSA	90.146	NSA	45.367	NSA	375.069	NSA
Total de hospitais no Apice ON	12	100,0	17	100,0	13	100,0	37	100,0	18	100,0	97	100,0

Fonte: SIH/DATASUS/CGSI.

Trat.: tratamento; trans. hip. grav. part. puerp.: transtorno hipertensivo na gravidez, parto e puerpério; NSA: não se aplica

A Tabela 4 apresenta as características dos nascimentos assistidos nesses hospitais. Há 75 (77,3%) hospitais com grande volume de partos (acima de 1.000), com destaque para o Norte, que tem 12 de seus 13 hospitais nessa categoria. Em relação a partos vaginais assistidos por enfermeiro obstetra, 85,6% dos hospitais têm uma proporção de 0 a 15%, com 94,1% dos hospitais do Nordeste nesse intervalo. Apenas seis (6,2%) estão acima de 50%, sendo quatro no Norte e dois no Sudeste. Para as taxas de cesárea, 32 hospitais (33,0%) estão entre 40% e 50%, 24 entre 50 e 60% e quinze acima de 60%. Chama a atenção que 39 HE (40,2%) apresentam percentuais acima de 50%, com destaque para 8 dos 12 hospitais (66,7%) do Centro-Oeste.

**Tabela 4:** Distribuição das características dos nascimentos assistidos nos hospitais integrantes do projeto Apice ON no Brasil e regiões de janeiro a junho de 2017.

Características	Centro-Oeste		Nordeste		Norte		Sudeste		Sul		Brasil	
	N	%	N	%	N	%	N	%	N	%	N	%
Volume de partos												
Até 499 partos	1	8,3	0	0,0	0	0,0	1	2,7	1	5,6	3	3,1
De 500 a 999 partos	3	25,0	2	11,8	1	7,7	11	29,7	2	11,1	19	19,6
Acima de 1.000 partos	8	66,7	15	88,2	12	92,3	25	67,6	15	83,3	75	77,3
Partos vaginais assistidos por enfermeiro obstetra												
Zero	2	16,7	0	0,0	1	7,7	15	40,5	8	44,4	26	26,8
De 0,01% a 5%	5	41,7	13	76,5	5	38,5	16	43,2	8	44,4	47	48,5
De 5% a 15%	3	25,0	3	17,6	2	15,4	0	0,0	2	11,1	10	10,3
De 15% a 50%	2	16,7	1	5,9	1	7,7	4	10,8	0	0,0	8	8,2
Acima de 50%	0	0,0	0	0,0	4	30,8	2	5,4	0	0,0	6	6,2
Baixo peso ao nascer												
Menor que 10%	2	16,7	3	17,6	6	46,2	4	10,8	4	22,2	19	19,6
De 10% a 15%	5	41,7	1	5,9	5	38,5	20	54,1	7	38,9	38	39,2
De 15% a 20%	4	33,3	4	23,5	1	7,7	7	18,9	4	22,2	20	20,6
Acima de 20%	1	8,3	9	52,9	1	7,7	6	16,2	3	16,7	20	20,6
Taxa de cesárea												
Até 30%	0	0,0	0	0,0	1	7,7	4	10,8	1	5,6	6	6,2
De 30% a 40%	0	0,0	1	5,9	2	15,4	11	29,7	6	33,3	20	20,6
De 40% a 50%	4	33,3	6	35,3	7	53,8	10	27,0	5	27,8	32	33,0
De 50% a 60%	7	58,3	6	35,3	2	15,4	5	13,5	4	22,2	24	24,7
Acima de 60%	1	8,3	4	23,5	1	7,7	7	18,9	2	11,1	15	15,5
Total de hospitais no Apice ON	12	100,0	17	100,0	13	100,0	37	100,0	18	100,0	97	100,0

Fonte: MS/SVS/DANTPS/CGIAE/Sinasc.

A Tabela 5 retrata as taxas de cesáreas nos hospitais do projeto em comparação com as dos hospitais do Brasil nesse primeiro semestre de 2017. Ao analisarmos o total de nascimentos no Brasil, encontrou-se 56,9% por cesárea, com maior proporção no Sul e Centro-Oeste (64,0% e 63,9%, respectivamente) e a menor no Norte (47,3%). Nos hospitais do projeto, a taxa foi de 42,0%, sendo maior no Centro-Oeste (49,1%) e menor no Sudeste (37,6%), evidenciando a grande diferença de prática cirúrgica entre os hospitais selecionados para o projeto comparados com os demais do país.

**Tabela 5:** Distribuição das taxas de cesáreas nos hospitais integrantes do projeto Apice ON no Brasil e regiões de janeiro a junho de 2017.

Procedimento	Centro-Oeste		Nordeste		Norte		Sudeste		Sul		Brasil	
	N	%	N	%	N	%	N	%	N	%	N	%
Brasil												
Cesárea	70.017	63,9	183.645	49,3	54.967	47,3	326.066	60,5	116.077	64,0	750.772	56,9
Total de partos	109.584	100,0	372.600	100,0	116.195	100,0	539.387	100,0	181.422	100,0	1.319.188	100,0
Apice ON												
Cesárea	7.138	49,1	16.850	47,8	14.339	41,2	20.596	37,6	10.233	40,2	69.156	42,0
Total de partos	14.542	100,0	35.269	100,0	34.780	100,0	54.732	100,0	25.460	100,0	164.783	100,0

Fonte: MS/SVS/DANTPS/CGIAE/Sinasc.

Nota: No cálculo dos hospitais do Brasil, foram excluídos os hospitais integrantes do projeto Apice ON. Pelo teste do qui-quadrado de Pearson (χ^2^), a diferença das proporções entre as regiões e tipo de parto foi estatisticamente significante para Brasil e Apice ON (p < 0,00001).

## DISCUSSÃO

Este estudo objetivou descrever a estrutura e alguns indicadores de processo dos hospitais integrantes do Apice ON, que são HE e têm papel fundamental para induzir mudanças do modelo vigente no país para um modelo que vem sendo construído desde os anos 2000, que objetiva uma assistência à mulher e ao recém-nascido humanizada e baseada em evidencias científicas, de forma a permitir que a mulher vivencie seu parto e o nascimento de seu filho como um processo natural, conforme preconiza a Organização Mundial de Saúde (OMS)^14^.

HE são caracterizados por serem extensão de estabelecimentos de saúde, realizarem práticas de ensino, serem reconhecidos oficialmente como de ensino e proporcionarem atendimento médico em nível terciário^15^. No Brasil, a Portaria Interministerial nº 1006/MEC/MS redefine o papel dos HE para atender às necessidades da população – dentre elas a humanização da atenção à saúde, orientada pela Política Nacional de Humanização –, implantando o cuidado integral^16^. Um estudo conduzido em 14 faculdades de medicina apontou que 86% da carga horária de estágios práticos ocorrem nos hospitais universitários e 14% nos demais estabelecimentos^17^. Portanto, eles devem ser modelares, para permitir aprendizagem sintonizada com as evidências científicas, assim como com as recomendações e normativas nacionais e internacionais de boas práticas.

Segundo a RC, espera-se do modelo humanista uma assistência contínua à parturiente, com uso de tecnologia adequada, evitando o uso de intervenções e técnicas invasivas sem indicação; tendo como local de parto, além de hospitais, CPNs; com a presença de enfermeiros obstetras responsáveis pelo acompanhamento do trabalho de parto sem distocia; e, caso seja identificada qualquer intercorrência, que eles possam encaminhar a parturiente para outros profissionais^11^.

Outro aspecto central da proposta é a adoção de boas práticas de atenção, dentre elas a garantia de acompanhante de escolha da mulher durante o trabalho de parto, parto e pós-parto imediato (PPP); práticas de gestão democrática e participativa nos serviços; ambiência adequada nos setores de obstetrícia e neonatologia, de acordo com a Resolução da Diretoria Colegiada (RDC) nº 36/2008 da Agência Nacional de Vigilância Sanitária (Anvisa), com centros de PN intra e peri-hospitalares; ambiência nos centros obstétricos, com ambientes individualizados para atenção continuada PPP, sem transferir a parturiente a outro ambiente; oferta de leitos obstétricos e neonatais gerais e especializados (UTI, canguru e Casa da Gestante, Bebê e Puérpera); e habilitação de Amigo da Criança, fortalecendo o início da amamentação e ofertando alojamento conjunto, entre outras práticas^18,19^.

Os achados deste estudo de linha de base revelaram que a maioria desses hospitais assiste gestações de alto risco, pois quase 95% dispõem de UTI neonatal, e mais de 80% têm importantes habilitações. Todavia, antes da implantação do projeto, a proporção da habilitação Amigo da Criança entre os hospitais era relativamente baixa (66%), embora esse programa exista desde 1994^20^; nove (9%) ainda não ofereciam alojamento conjunto, recomendado desde os anos 1980^21^; poucos ofereciam o método canguru (45,4%), cuja primeira portaria data de 2000^22^; era quase nula proporção de hospitais com Casa da Gestante, Bebê e Puérpera (3,1%); e a maioria tinha sala de pré-parto (97%) e para PN (93%) separadas, divergindo da RDC nº 36/2008 da Anvisa^19^; havia poucos enfermeiros obstetras (1% do total de profissionais cadastrados no CNES), e a proporção de partos assistidos por eles foi superior a 50% dos partos vaginais apenas em seis hospitais, ficando entre 15 e 50% em outros oito; a taxa de cesáreas média foi de 42%, variando entre 37,6% (Sudeste) e 49,1% (Nordeste); ademais, embora a Lei nº 11.108^23^ e a Portaria GM/MS nº 2418 que a regulamentou^24^ sejam de 2005, para dez hospitais não constava cobrança de diária de acompanhante na AIH.

Portanto, apesar de a RC ser de 2011, objetivando a mudança do modelo de atenção para o humanista, e apesar de o Programa de Humanização do Pré-natal e Nascimento ter sido instituído em 2000, constata-se que, em 2017, a maioria dos HE ainda adotava e reproduzia a ambiência e práticas do modelo tecnocrático, tendo em vista suas taxas de cesáreas e as baixas proporções de partos vaginais assistidos por enfermeiro obstetra.

É importante lembrar que em 1996 a OMS editou uma publicação^25^ que classificava as práticas de atenção ao PN em quatro categorias, com base no conhecimento científico disponível à época: A. práticas comprovadamente úteis e que devem ser estimuladas; B. práticas claramente prejudiciais ou ineficazes e que devem ser eliminadas; C. práticas sem evidências suficientes para apoiar uma recomendação clara e que devem ser utilizadas com cautela até que mais pesquisas esclareçam a questão; e D. práticas frequentemente usadas de modo inadequado. Essas recomendações foram atualizadas em 2018, adotando um novo formato^14^.

Não foi possível identificar a adoção das práticas relacionadas na publicação da OMS de 1996, havendo informações apenas sobre cesarianas. Com relação ao país, o inquérito Nascer no Brasil apontou que, quanto às boas práticas em mulheres com risco habitual, 44,3% referiram movimentação durante o trabalho de parto (categoria A). Em 74,9% dos partos houve uso de cateter venoso central (categoria B), em 39,1% foi realizada amniotomia (categoria C) e em 36,4% foi utilizada a ocitocina (categoria C). Em relação ao parto vaginal, 91,7% ficaram na posição de litotomia (categoria B) e 53,5% foram submetidas à episiotomia (categoria D). Quanto ao tipo de parto, 51,9% tiveram cirurgias cesarianas (categoria D) e apenas 5% tiveram parto vaginal sem nenhuma intervenção durante todo o trabalho de parto e parto^26^.

Quanto às práticas de atenção ao recém-nascido saudável, no Brasil, 71,0% tiveram suas vias aéreas superiores aspiradas, não recomendada na atualização de 2018^14^. Apenas 28,2% tiveram contato pele a pele ao nascer (categoria A), 69% ficaram em alojamento conjunto (categoria A) e apenas 44,5% mamaram na primeira hora de vida (categoria A)^27^.

Pode-se supor que esses dados nacionais sejam similares aos dos HE. Constata-se, assim, o seu papel estratégico para que ocorra mudança do modelo tecnocrático de atenção para o humanista pois, se o ensino ocorre em locais que adotam este modelo, assim como práticas cientificamente fundamentadas, será mais provável que os profissionais as reproduzam em suas futuras práticas. Rattner^5^ (2009) considerou paradoxal que, no Brasil, práticas de atenção perinatal baseadas em evidências científicas, preconizadas pela OMS e MS, não fossem adotadas em instituições de ensino e que, ao contrário, fossem um dos focos de resistência à sua adoção, uma vez que se espera que a academia seja o *locus* privilegiado para o avanço do conhecimento e uso das melhores práticas. Hotimsky^28^, em estudo para análise da formação em obstetrícia em duas escolas renomadas de medicina, procurou identificar como se unem a competência técnico-científica e o cuidado com a mulher no ensino teórico-prático de assistência ao parto. Entre seus vários achados, destacam-se: não compartilhamento, com as mulheres, das decisões sobre condutas a serem realizadas; presença de acordos informais entre equipes para escala de plantões; predomínio da transmissão oral do conhecimento técnico-científico, desvalorizando a transmissão de valores humanistas e desconsiderando evidências científicas recentes para definir as práticas.

Mudanças nas diretrizes curriculares nacionais das disciplinas em saúde vêm sendo propostas desde 2001. Dentre as propostas para o modelo biomédico, insere-se o ensino da humanização para a assistência integral e melhoria no cuidado. Contudo, somente a inserção de conteúdos e disciplinas envolvendo o tema não garantem relevantes mudanças na formação, posto que esses conteúdos são dispersos em disciplinas que pouco se comunicam entre si. Humanização é muito mais que um conteúdo, pois abarca aspectos filosóficos da formação, práticas docentes e atitudes profissionais nos cenários da saúde e educação, que se configuram como modelo para a prática futura, visto que professores são modelos de papel social e profissional durante a graduação^29^.

As principais limitações deste estudo derivam do uso de dados secundários, com possibilidade de incompletude, sub-registro e registro inadequado das informações. É possível que alguns indicadores não reflitam a realidade da ambiência, já que nem todas instituições atualizam seus dados periodicamente no CNES. O mesmo se aplica às AIH e declarações de nascido vivo, embora o viés destas provavelmente seja menor, uma vez que o SIH/SUS serve para faturamento e há boa completude para as variáveis do Sinasc aqui utilizadas. Outra importante limitação é dada pelo fato de o projeto Apice ON ser inédito, ou seja, há carência de publicações sobre projetos semelhantes para enriquecer esta discussão. Por outro lado, estudos como este são imprescindíveis pois, ao realizar diagnósticos de linha de base, produzem suporte necessário para avaliar futuramente o impacto das ações do Apice ON nos HE, subsidiando futuras decisões. Ademais, o próprio fato de utilizar bases de dados secundárias torna factível um processo de monitoramento desses mesmos indicadores sem investimentos maiores.

Enfim, o cenário de mudança revela-se promissor, uma vez que há grande distância entre o que deveria ser adotado e ensinado nessas instituições e o que este retrato de linha de base evidencia. Para Davis-Floyd et al. (p. 452)^30^:

Provedores de cuidado tendem a oferecer assistência como a aprenderam, de modo que, quando lhes são apresentadas novas informações, muitos com longa trajetória se recusam a integrá-las ou implantá-las porque estão muito habituados a atuar como seus mestres agiam. Um ponto crucial para a criação e manutenção de modelos de atenção ao parto e nascimento que funcionam é a reforma da educação profissional, para que, em vez de serem educados no modelo biomédico orientado para tecnologias e patologias, estudantes de medicina, enfermagem e parteria (*midwifery*) sejam educados no modelo de atenção a partos e nascimentos orientado para a normalidade e parteria. [Tradução livre das autoras].

Hotimsky^28^ identificou, em 2007, que o ensino nas instituições estudadas ainda não se havia apropriado de conteúdos cientificamente embasados e das práticas humanizadas de atenção perinatal. Pode-se supor, a partir destes resultados, que o cenário encontrado nos HE em 2017 (ou seja, mais de vinte anos após a publicação da OMS) permanece inalterado, o que comprova a pertinência do Apice ON como estratégico para efetivar a política pública representada pela RC, que se propõe a induzir mudança do modelo de atenção nos HE. Espera-se que um estudo similar futuro identifique grandes mudanças ocorridas nessas instituições, seja na ambiência e outros aspectos da estrutura, seja no processo de cuidado, destarte impactando nos seus resultados, mas, com maior propriedade, na prática profissional dos futuros profissionais de saúde.
